# Association of BRI With Psoriasis and Mediator Effect of SII: A Study Based on NHANES (2003–2006, 2009−2014)

**DOI:** 10.1155/mi/7953174

**Published:** 2026-03-06

**Authors:** Ningxin Zhang, Jiaqi Li, Ping Song

**Affiliations:** ^1^ Department of Dermatology, Xiyuan Hospital of China Academy of Chinese Medical Sciences, Haidian District, Beijing, 100091, China, cacms.ac.cn

**Keywords:** BRI, mediator effect, NHANES, psoriasis, SII

## Abstract

**Background and Aims:**

The prevalence of psoriasis (Pso), a chronic immune‐mediated inflammatory skin disease, is high in patients with obesity. This study investigates the mediating role of the systemic immune‐inflammation index (SII) in the association between body roundness index (BRI) and Pso prevalence.

**Methods and Results:**

This study included 14,669 participants from the National Health and Nutrition Examination Survey (NHANES) (2003–2006, 2009−2014). The presence or absence of obesity was identified using the BRI, and Pso was assessed using the Pso questionnaire. The association of BRI with Pso was explored by weighted multivariate logistic regression analyses and restricted cubic spline (RCS) analyses, and the threshold effect was further examined using two‐stage linear regression models. Mediation analyses assessed SII’s role in the BRI‐Pso association. The Pso group included 445 out of 14,669 participants. Logistic regression analyses indicated a positive association between BRI and Pso after adjusting for potential confounders. RCS analyses showed a nonlinear relation between BRI and Pso (*P*_nonlinear_ = 0.018), with a point of inflection at 5.103 (odds ratio [OR] = 1.09, 95% CI: 1.04, 1.14). BRI and Pso were positively correlated on the left side of the point of inflection (OR = 1.25, 95% CI: 1.09, 1.45), whereas such an association was not detected on the right side (OR = 1.04, 95% CI: 0.97, 1.11). Mediation analysis showed SII partially mediated this association, accounting for 9.48% of the effect (*p* < 0.001).

**Conclusion:**

BRI is positively associated with Pso, with SII playing a mediating role. These findings highlight the importance of visceral fat management and SII monitoring in addressing Pso and its metabolic comorbidities.

## 1. Introduction

Psoriasis (Pso) is a chronic immune‐mediated inflammatory skin disease affecting ~125 million worldwide population [1]. It places a heavy burden on society and patients’ health and psychology due to systemic erythema, scales, and inflammatory papules [2], with anxiety and suicidal tendencies among 57% of patients [3]. In addition to the skin manifestations, the risk of obesity, diabetes, hyperlipidemia, and cardiovascular disease is also increased by Pso [4, 5], a systemic disease [4]. Pso has an unknown pathogenesis and a complex mechanism, and it is currently considered to result from the interaction of immunity, metabolism, environment, and emotion in the context of polygenic inheritance [6]. Recently, Pso has been treated primarily with biological agents, retinoic acid, immunosuppressants, topical glucocorticoids, and vitamin D3 derivatives, achieving better effects. However, these therapies have high costs, unstable efficacy, and poor tolerability [1].

Increasingly more epidemiologic research has been conducted on the association of Pso with obesity. We have found a global prevalence of 25% for comorbid Pso and obesity, specifically 18% in children and adolescents and 35% in adults [7]. Recently, obesity has been recognized as an independent risk factor for the onset and severity of Pso [8]. Obesity may play a role during Pso onset and progression through inflammatory factors, interference with immunity and metabolism by influencing microbiota [9], and even nonmetabolic effects (e.g., mechanical effects) [10]. Body roundness index (BRI) is a new measure of obesity, taking into account the ratio of waist circumference (WC) to height rather than weight [11]. Compared with traditional waist measurement, BRI presents a more accurate picture of the distribution of abdominal fat and the accumulation of visceral fat [12].

The systemic immune‐inflammation index (SII) is an innovative inflammatory biomarker based on immune cell subsets and platelet counts, which can reflect local immune responses and systemic inflammatory responses [13]. Recently, the application of SII has been expanded, and a growing body of research has shown that SII can also be used to predict the severity of some diseases (including cancer, metabolic disorders, and inflammation [14–17] and the therapeutic effect. Pso is a chronic inflammatory skin disease closely related to a variety of inflammatory cytokines (e.g., IL‐17 and TNF‐α) [18]. SII has a significantly higher value in Pso patients and an association with activation and severity of Pso [19–21].

In general, Pso is a stubborn and intractable systemic disease manifested as erythema and scales on the skin. Some possible associations of obesity and SII with Pso have been identified in several studies, but the mediator effect of SII in the association of BRI with Pso remains to be verified. This study intends to explore the mediator effect of SII between BRI and Pso prevalence based on the National Health and Nutrition Examination Survey (NHANES).

## 2. Materials and Methods

### 2.1. Data Source and Participant Selection

Conducted by the National Center for Health Statistics (NCHS), NHANES is a comprehensive survey specifically designed to assess the health and nutritional status of civilians in the United States by collecting and analyzing health data from nationally representative samples. NHANES contains a wealth of health information, including body measurement data (e.g., height, weight, and blood pressure), laboratory test results (e.g., analysis results of blood and urine samples), health questionnaires (covering dietary habits, lifestyle, and health status), and nutritional assessment. With these data, researchers can have access to valuable information resources to gain insight into the health status and nutritional demand of the US population, offering a scientific basis for policy development and public health decision‐making. The NHANES protocol received approval by the NCHS Ethics Review Board, and all participants gave written informed consent.

**Figure 1:**
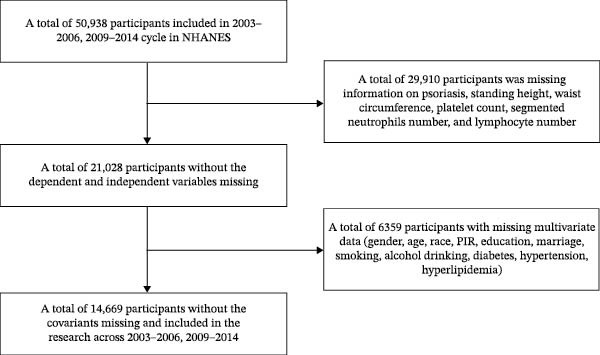
Flowchart of participant selection from NHANES (2003–2006, 2009−2014).

We utilized data from 50,938 participants from the NHANES (2003–2006, 2009−2014). Then 29,910 participants were excluded due to missing data on Pso, standing height, WC, platelet count, neutrophil count, and lymphocyte count, and 6359 participants were excluded due to lack of covariates (gender, age, race, PIR, educational level, marital status, smoking, drinking, diabetes, hypertension, and hyperlipidemia). Ultimately, 14,669 participants were included (Figure 1).

### 2.2. Ascertainment of Pso

The presence or absence of Pso was ascertained using the Dermatology Questionnaire Section (DEQ)—DEQ053 “Ever told had Psoriasis?” of the NHANES (2003–2006) and the Medical Conditions Questionnaire (MCQ)—MCQ070 “Ever been told you have psoriasis?” of the NHANES (2009–2014). Participants who answered “Yes” to DEQ053 or MCQ070 were considered to have Pso, and those who answered “No” were considered to not have Pso.

### 2.3. Ascertainment of BRI

BRI, a weight‐independent measure, was calculated using the model proposed by Thomas et al. [11]: BRI = 364.2 – 365.5 × (1 − [WC (m) / 2π]^2^ / [0.5 × height (m)]^2^)^½^. BRI outperforms body mass index (BMI) in predicting the body fat rate and visceral fat volume [11]. Participants’ standing height and WC were extracted from the BMXHT and BMXWAIST results of the Body Measures Questionnaire.

### 2.4. Ascertainment of SII

SII is a composite measure calculated as SII = *P*× *N* /*L*, where *P*, *N*, and *L* stand for platelet count, neutrophil count, and lymphocyte count, respectively [22]. *P*, *N*, and *L* were extracted from the LBXPLTSI, LBDNENO, and LBDLYMNO results of the Complete Blood Count with Five‐Part Differential—Whole Blood Questionnaire (L25, CBC).

### 2.5. Covariates

Covariates included demographic characteristics (gender, age, and race: Mexican American, other Hispanic, non‐Hispanic White, non‐Hispanic Black, and other), socioeconomic indicator (PIR: the higher, the better the family economic conditions [23, 24]), educational level (<high school, >high school, high school/equivalent), marital status (married/cohabiting, unmarried, widowed/divorced/separated), smoking (never/former/current), drinking (over 12 cups of alcoholic beverages consumed per year or not), and diseases (diabetes, hypertension, and hyperlipidemia). Diabetes was defined as either diagnosis or treatment of hyperglycemia, with hemoglobin A1c ≥1cth hemoglobin A1c as eith ≥1cth hedL, or a 2 h blood glucose ≥ or a 2 dL [25, 26]. Participants were considered to have hypertension if they met any of the following criteria: (1) They answered “Yes” to the question about whether they had hypertension in the questionnaire. (2) They reported the use of antihypertensive medications [27, 28]. (3) Three consecutive blood pressure readings from the NHANES section and the average systolic and diastolic blood pressure: an average systolic blood pressure of 130 mmHg or higher or an average diastolic blood pressure of 80 mmHg or higher (after sitting quietly for 5 min and determining the maximum inflation level) [29]. Hyperlipidemia was diagnosed based on a doctor’s judgment.

### 2.6. Statistical Analysis

Based on the results of the Pso questionnaire, participants were assigned into Pso and non‐Pso groups. Continuous variables were tested for normality. Normally distributed continuous variables were described by mean ± standard deviation (SD), and nonnormally distributed ones were described by median and quartiles [M (Q1, Q3)] and compared between two groups by *t*‐tests or Kruskal–Wallis *H*‐tests. To account for the complex sampling design of the NHANES, we applied the appropriate sample weights provided by the NHANES. The association of BRI with Pso was explored by multivariate logistic regression analyses, with odds ratio (OR) and 95% confidence interval (CI) calculated. Model 1 (a crude model) was not adjusted, Model 2 was adjusted for demographic characteristics (gender, age, and race) [30] and PIR, and Model 3 was further adjusted for educational level, marital status, smoking, drinking, and disease (diabetes, hypertension, and hyperlipidemia). In these models, trend tests were also conducted when BRI was considered an ordered four‐category variable. In addition, restricted cubic spline (RCS) analyses were performed for the nonlinear relation between BRI and Pso. We assessed the potential nonlinear association between exposure and outcome using an RCS with five knots at the 5th, 27.5th, 50th, 72.5th, and 95th percentiles. *P*‐nonlinear values are shown in the corresponding figures. The presence or absence of heterogeneity and interactions in specific populations was examined by subgroup analyses on gender, race, educational level, marital status, smoking, drinking, and disease (diabetes, hypertension, and hyperlipidemia).

The mediator effect of SII was determined by mediation analyses. First, a fully adjusted regression model was adopted to investigate the effect of BRI on SII and the effect of SII on Pso, identifying the potential of SII as a mediator between BRI and Pso. Subsequently, mediation analyses were conducted using the R mediation package to assess the SII‐mediated indirect, direct, and overall effects of BRI on Pso. The proportion of SII mediation was determined by dividing the indirect effect by the total effect.

R4.4.1 was utilized for statistical analyses. *p* ≤ 0.05 (two‐sided) was deemed statistically significant.

## 3. Results

### 3.1. Basic Information

This study included 14,669 participants (7083 males [48.3%] and 7586 females [51.7%]), including 445 (3.03%) in the Pso group and 14,224 (96.97%) in the non‐Pso group. The participants’ median age and median PIR were 46.0 years (IQR: 35.0–57.0) and 3.28 (IQR: 1.62–5.00), respectively. Participants were categorized by race as non‐Hispanic white: 7075 (48.2%), non‐Hispanic black: 3079 (21.0%), Mexican American: 1862 (12.7%), other Hispanic: 1167 (8.0%), and other races: 1486 (10.1%). For the educational level, there were 2929 (20.0%) cases <high school, 3164 (21.5%) cases of high school/equivalent, and 8576 (58.5%) cases > high school. The marital status was categorized into married/cohabiting: 9063 (61.8%), unmarried: 2544 (17.3%), and widowed/divorced/separated: 3062 (20.9%). There were 8126 (55.4%) never‐smokers, 3567 (24.3%) former smokers, and 2976 (20.3%) current smokers. 10,746 (73.3%) participants consumed over 12 cups of alcoholic beverages per year. Diabetes, hypertension, and hyperlipidemia occurred in 2476 (16.9%), 7742 (52.8%), and 5432 (37.0%) participants, respectively. Significant differences were found in all characteristics except gender, PIR, drinking, and diabetes between the two groups (*p* < 0.05) (Table 1).

**Table 1:** Weighted characteristics of participants.

Variable	Total (*n* = 14,669)	Non‐Pso (*n* = 14,224)	Pso (*n* = 445)	*p*
Gender, *n* (%)				0.13
Male	7083 (48.3)	6856 (48.2)	227 (51.0)	—
Female	7586 (51.7)	7368 (51.8)	218 (49.0)	—
Age (years)	46 (35, 57)	46 (35, 57)	50 (39, 58)	0.004
Race, *n* (%)				<0.001
Mexican American	1862 (12.7)	1828 (12.9)	34 (7.7)	—
Other Hispanic	1167 (8.0)	1131 (8.0)	36 (8.1)	—
Non‐Hispanic white	7075 (48.2)	6800 (47.8)	275 (61.8)	—
Non‐Hispanic black	3079 (21.0)	3021 (21.2)	58 (13.0)	—
Other race	1486 (10.1)	1444 (10.1)	42 (9.4)	—
PIR	3.28 (1.62, 5.00)	3.28 (1.61, 5.00)	3.45 (1.75, 5.00)	0.2
Educational level, *n* (%)				0.3
<High school	2929 (20.0)	2851 (20.0)	78 (17.5)	—
>High school	8576 (58.5)	8303 (58.4)	273 (61.4)	—
High school/equivalent	3164 (21.5)	3070 (21.6)	94 (21.1)	—
Marital status, *n* (%)				0.8
Married/cohabiting	9063 (61.8)	8790 (61.8)	273 (61.3)	—
Unmarried	2544 (17.3)	2480 (17.4)	64 (14.4)	—
Widowed/divorced/separated	3062 (20.9)	2954 (20.8)	108 (24.3)	—
Smoking, *n* (%)				<0.001
Never	8126 (55.4)	7927 (55.7)	199 (44.7)	—
Former	3567 (24.3)	3413 (24.0)	154 (34.6)	—
Current	2976 (20.3)	2884 (20.3)	92 (20.7)	—
Drinking, *n* (%)				0.7
No	3923 (26.7)	3808 (26.8)	115 (25.8)	—
Yes	10,746 (73.3)	10,416 (73.2)	330 (74.2)	—
Diabetes, *n* (%)				0.5
No	12,193 (83.1)	11,839 (83.2)	354 (80.0)	—
Yes	2476 (16.9)	2385 (16.8)	91 (20.0)	—
Hypertension, *n* (%)				<0.001
No	6927 (47.2)	6756 (47.5)	171 (38.4)	—
Yes	7742 (52.8)	7468 (52.5)	274 (61.6)	—
Hyperlipidemia, *n* (%)				0.004
No	9237 (63.0)	9003 (63.3)	234 (52.6)	—
Yes	5432 (37.0)	5221 (36.7)	211 (47.4)	—
SII	481 (349, 675)	478 (348, 670)	545 (383, 765)	<0.001
Platelet count (1000 cells/μL)	240 (204, 285)	240 (204, 285)	240 (211, 290)	0.2
Segmented neutrophil count (1000 cells/μL)	4.00 (3.10, 5.20)	4.00 (3.10, 5.10)	4.30 (3.20, 5.50)	0.015
Lymphocyte count (1000 cells/μL)	2.00 (1.60, 2.50)	2.00 (1.60, 2.50)	1.90 (1.50, 2.40)	0.085
BRI	4.93 (3.73, 6.48)	4.91 (3.71, 6.46)	5.33 (4.14, 6.90)	<0.001
Standing height (cm)	169 (162, 177)	169 (162, 177)	171 (163, 177)	0.076
Waist circumference (cm)	98 (88, 109)	98 (87, 109)	102 (93, 112)	<0.001

### 3.2. Association Between BRI and Pso

The results of the trend test indicated that as BRI rose, the Pso risk displayed a statistically significant increasing trend (*p* < 0.05) in Model 1 (OR: 1.09, 95% CI: 1.05, 1.14), Model 2 (OR: 1.10, 95% CI: 1.05, 1.15), and Model 3 (OR: 1.09, 95% CI: 1.04, 1.14). After adjusting for all covariates, a per unit increase in BRI was linked to a 9.0% rise in the Pso risk. In Model 3, the second quartile Q2 (OR: 1.55, 95% CI: 0.99, 2.44) had no statistically significant difference from Q1 in the reference group, but Q3 (OR: 1.72, 95% CI: 1.24, 2.40) and Q4 (OR: 1.94, 95% CI: 1.29, 2.93) were significantly different, suggesting that the Pso risk elevated significantly as BRI increased (*p* for trend = 0.005) (Table 2).

**Table 2:** Association between BRI and Pso.

Participant	Model 1		Model 2		Model 3	
	OR (95% CI)	*p*	OR (95% CI)	*p*	OR (95% CI)	*p*
BRI						
Continuous	1.09 (1.05, 1.14)	<0.001	1.10 (1.05, 1.15)	<0.001	1.09 (1.04, 1.14)	0.001
Tertiles						
Q1	Ref		Ref		Ref	
Q2	1.67 (1.08, 2.58)	0.023	1.62 (1.04, 2.53)	0.034	1.55 (0.99, 2.44)	0.055
Q3	1.89 (1.38, 2.59)	<0.001	1.85 (1.34, 2.55)	<0.001	1.72 (1.24, 2.40)	0.002
Q4	2.11 (1.48, 3.01)	<0.001	2.16 (1.47, 3.18)	<0.001	1.94 (1.29, 2.93)	0.002
*p* for trend	<0.001	—	<0.001	—	0.005	—

**Figure 2:**
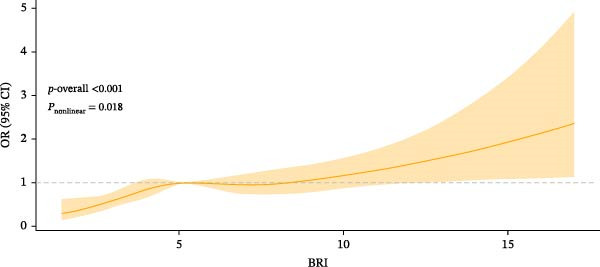
Fitting model between BRI and Pso. The inflection point of the RCS curve was located at 5.103 (log‐likelihood ratio *p* = 0.022). Adjusted variables: gender, age, race, educational level, marital status, PIR, smoking, drinking, diabetes, hypertension, and hyperlipidemia.

To further visualize the association of BRI with Pso, RCS analyses were conducted, and a nonlinear relation between BRI and Pso was confirmed (*P*_nonlinear_ = 0.018) (Figure 2). Furthermore, the threshold effect was examined using two‐stage linear regression models (Table 3). The point of inflection of the RCS curve was 5.103 (log‐likelihood ratio *p* = 0.022), i.e., the Pso risk elevated by 25% for each unit increase in BRI when BRI was below 5.103, while such an association vanished when BRI was above 5.103. It can be seen that further increases in BRI did not significantly increase the Pso risk.

**Table 3:** Threshold effect between BRI and Pso.

BRI	OR (95% CI)	*p*
Continuous	1.09 (1.04, 1.14)	<0.001
Point of inflection		
BRI < 5.103	1.25 (1.09, 1.45)	0.002
BRI ≥ 5.103	1.04 (0.97, 1.11)	0.200
Log‐likelihood ratio *p*	0.02172	<0.001

### 3.3. Subgroup Analyses

**Figure 3:**
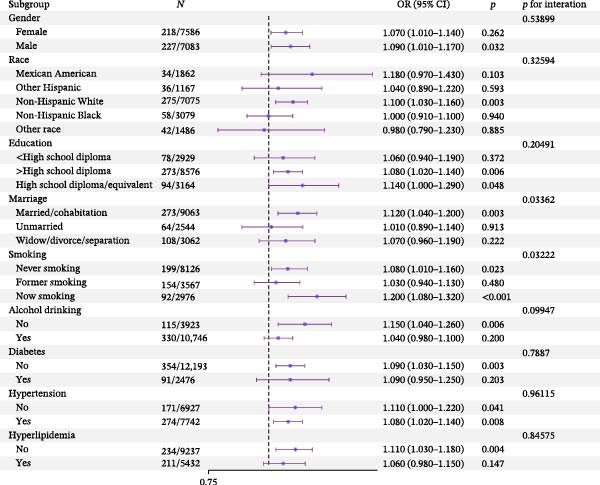
Forest plot of subgroup analyses of BRI and Pso.

We conducted risk stratification analyses on BRI across subgroups defined by gender, race, smoking, drinking, diabetes, hypertension, and hyperlipidemia. In the following subgroups, BRI was significantly associated with an increased Pso risk: male (OR: 1.09, 95% CI: 1.01, 1.17), non‐Hispanic white (OR: 1.10, 95% CI: 1.03, 1.16), >high school (OR: 1.08, 95% CI: 1.02, 1.14), married/cohabiting (OR: 1.12, 95% CI: 1.04, 1.20), smoker (OR: 1.20, 95% CI: 1.08, 1.32), never‐smoker (OR: 1.08, 95% CI: 1.01, 1.16), no drinking (OR: 1.15, 95% CI: 1.04, 1.26), no diabetes (OR: 1.09, 95% CI: 1.03, 1.15), hypertension (OR: 1.08, 95% CI: 1.02, 1.14), no hypertension (OR: 1.09, 95% CI: 1.03, 1.15), and no hyperlipidemia (OR: 1.11, 95% CI: 1.03, 1.18). However, except for marital status (*p* = 0.034) and smoking (*p* = 0.032), no interaction was observed between these variables and BRI (*p* for all interactions > 0.05) (Figure 3).

### 3.4. Mediation Analyses

**Figure 4:**
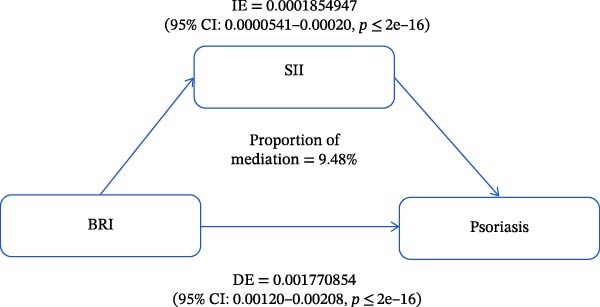
Estimated proportion of SII medication in the association of BRI with Pso. DE, direct effect; IE, indirect effect.

BRI, SII, and Pso served as independent, mediator, and dependent variables, respectively, in the mediation analyses, and the mediation model and path are shown in Figure 4. We found a significant direct effect of BRI on Pso (DE = 0.001770854, 95% CI: 0.00120–0.00208, *p*≤ 2e–16), and further observed that BRI had a significant indirect effect on Pso, which was mediated by SII (IE = 0.0001854947, 95% CI: 0.0000541–0.00020, *p*≤ 2e–16). To sum up, SII partially mediated the association of BRI with Pso, with a proportion of mediation of ~9.48% (*p* < 0.001).

## 4. Discussion

In this study, we found a nonlinear positive relation between BRI and Pso, and the subgroup analyses showed that male, non‐Hispanic white, >high school, married/cohabiting, smoker, never‐smoker, no drinking, no diabetes, hypertension, no hypertension, and no hyperlipidemia subgroups correlated with Pso. Among them, marital status and smoking were significantly associated with BRI, with interaction effects. In addition, SII partially mediated the association of BRI with Pso, with a proportion of mediation of 9.48% (*p* < 0.001).

Pso is an immune‐mediated chronic inflammatory disease that affects the whole body, and almost all sites of secondary inflammation are dependent on the severity of Pso and systemic therapeutic regimens [31]. The proportion of Pso patients with obesity is significantly higher, and a multifactor correlation between obesity and Pso has been verified by several studies, in which dietary habits, lifestyle, some genetic factors, and microbiota are the major factors during the progression of the two. Moreover, obesity is an independent risk factor for Pso, directly raising the Pso risk [8], which is associated with chronic pro‐inflammatory states [32]. BRI is a new measure for obesity that can reflect fat distribution more intuitively. Meanwhile, dyslipidemia is a key feature of metabolic syndromes in patients with obesity, which has been widely described in the literature with a consensus [33, 34]. Obesity can raise the concentration of saturated fatty acids (SFAs). First, SFAs can directly stimulate the expression of pro‐inflammatory cytokines [35], activate Toll‐like receptors, bind to cytoplasmic epidermal fatty acid‐binding proteins, activate retinoic acid receptors, stimulate the differentiation of CD11c^+^ macrophages [36, 37], and modulate ceramide synthesis [38]. Second, SFAs amplify pro‐inflammatory responses in the presence of danger signals. SFAs stimulate the expression of pro‐inflammatory cytokines by binding to peroxisome proliferator‐activated receptors, activating inflammasomes, and regulating ceramide synthesis [39]. At the gene level, people will become susceptible to Pso when the expression of genes directly related to inflammation, such as IL‐1, IL‐6, IL‐8 [40], IL‐17, IL‐23, and IL‐2 [41] is altered, many of which also experience changes in obesity. In addition, obesity weakens the efficacy of traditional medications and increases adverse drug reactions [42, 43]. GLP‐1 analogs exhibit significant effects of weight loss and anti‐inflammatory properties, so they may ameliorate low‐grade systemic inflammation associated with obesity, contributing to the Pso treatment. This also indirectly corroborates the strong association of BRI, Pso, and SII [44–46].

In this study, the subgroup analyses revealed significant interactions of marital status and smoking with susceptibility to Pso (BRI). A review showed that single men and women’s transitioning to marriage or cohabitation is associated with weight gain [47]. Smoking is an environmental risk factor for Pso [18, 48], and a high proportion of smokers in a Pso cohort was observed in a cross‐sectional study [48]. Research suggests that Pso patients have significantly increased serum MDA levels and decreased SOD levels and that smokers have significantly lower serum MDA levels and a significantly higher Pso area and severity index (PASI) than nonsmokers. Therefore, it can be inferred that smoking may contribute to the onset and exacerbation of Pso by inducing oxidative stress [49].

In addition, metabolic diseases have a significant impact on the incidence of Pso. Individuals with hypertension experience a higher incidence of Pso, while those without diabetes and hyperlipidemia have a lower incidence of Pso. Besides, hypertension and hyperlipidemia have also been identified as comorbidities of Pso by several studies [1, 50, 51], consistent with the findings of this study.

This study covered the largest number of participants (14,669 Americans from NHANES) to date examining the association of BRI with Pso. Few previous studies are available on the association between the two, and only one cross‐sectional study was conducted on BRI and Pso in Americans and found a positive correlation [52] (Genlong Bai), consistent with the findings of this study. Some of the discrepancies in covariates and subgroup analyses between the two studies can be attributed to the following reasons: First, the covariates in this study differed from those in the study by Genlong Bai. Although both studies took gender, race, PIR, educational level, marital status, smoking, drinking, hypertension, and diabetes as covariates, this study further categorized smoking as never/former/current smokers, while Genlong Bai only categorized it as smoking and nonsmoking. Moreover, the prevalence of hyperlipidemia was also incorporated in this study, so the models adjusted for the covariates made different conclusions. Second, this study also investigated SII as a mediator in the association of BRI with Pso for the first time, so that we can estimate the proportion of mediation and the intrinsic association between the two.

This study has the following strengths. First, obesity is currently evaluated almost exclusively based on BMI [53]. Several studies have demonstrated that BMI has a strong association with Pso [54, 55], and the Pso risk is lowest when BMI is 20, and it significantly increases as BMI rises. However, BMI cannot reflect the fat distribution in the body, especially the accumulation of abdominal fat, which is closely linked to the risk of multiple metabolic diseases. BRI is more scientific than BMI in the evaluation of obesity and outperforms BMI in evaluating visceral fat volume and predicting all‐cause mortality [56]. Compared with B‐mode ultrasound of the liver and gallbladder, CT, and MRI, which require complex diagnostic equipment or invasive evaluation of visceral fat, BRI is more simple, convenient, and noninvasive. Moreover, BRI can be measured in the case of insufficient basic equipment, so it possesses greater clinical utility and is economically feasible [11]. Second, the weighted national samples from the NHANES were utilized to better reflect the association of BRI with Pso in US adults. Third, previous studies were deeply reviewed, and several cross‐sectional studies were consulted to fully take into account potential confounders in the association between the two. Multivariate regression models and RCS fitting models were utilized to draw more accurate conclusions. Fourth, mediation analyses were conducted on SII in the association between obesity and Pso, which was the first joint analysis of the three.

Several limitations are worth noting. First, this observational study could not demonstrate the causality between BRI and Pso. Second, although many potential confounders were adjusted, the impact of unmeasured confounders on the results could not be eliminated. Third, IL‐17, IL‐23, and TNF‐α are often used to evaluate the degree of inflammation in Pso [57], but records on these indicators are deficient in the NHANES. SII does not directly evaluate humoral immunity or cellular immunity, but the changing trend of the immune status can be inferred based on the SII variation, and whether SII can serve as a major measure for the degree of inflammation in Pso remains to be further explored. In the future, we can conduct a longitudinal study as well as experimental validation of the association between BRI and Pso and determine by clinical studies whether BRI is superior to BMI in the measurement of obesity and whether SII can represent the inflammatory state in Pso. With improvements in these indicators, Pso sample collection and assessment of severity in areas with backward medical technology can be assisted, and guidance can be offered to treatment and prognosis. In addition to inflammation, other mediators, such as intestinal flora disorders and the proportion of mediation, can also be studied. Finally, this study utilized the NHANES data and relied on the data from US adults, restricting the generalizability of the findings to other populations.

In conclusion, the association of BRI with Pso was investigated in this study. We found a nonlinear positive relation between BRI and Pso, and SII was an important mediator in the association of BRI with Pso, suggesting that SII monitoring can effectively mitigate the effect of obesity on Pso.

## Author Contributions

All authors contributed to the study conception and design. Ningxin Zhangand Ping Song were involved in the conceptualization of the study, supervision, and manuscript editing.Ningxin Zhang carried out the majority of the experiments, data analysis, and wrote the manuscript in consultation with Ping Song. Jiaqi Li contributed to supervision and formal analysis. Ping Song was responsible for study funding and resources.

## Funding

This work was supported by the Dengfeng Project of the Beijing High‐level Innovation and Entrepreneurship Talent Support Program (Grant G202514020) and General Project of National Natural Science Foundation of China (Grant 82474521).

## Disclosure

All authors read and approved the final manuscript.

## Ethics Statement

All methods in our research were performed in accordance with the Declaration of Helsinki. This study was based on a public database and approved by the Research Ethics Review Committee of the National Center for Health Statistics (NCHS).

## Consent

All participants gave written informed consent, and ethical review was exempted in this study since it utilized publicly available NHANES data.

## Conflicts of Interest

The authors declare no conflicts of interest.

## Data Availability

The datasets analyzed during the current study are available in the National Center for Health Statistics (NCHS), https://www.cdc.gov/nchs/nhanes/about_nhanes.htm.

## References

[1] Armstrong A. W. and Read C. , Pathophysiology, Clinical Presentation, and Treatment of Psoriasis: A Review, JAMA. (2020) 323, no. 19, 1945–1960, 10.1001/jama.2020.4006. 32427307

[2] Bulat V. , Šitum M. , Delaš Aždajić M. , Lovrić I. , and Dediol I. , Study on the Impact of Psoriasis on Quality of Life: Psychological, Social and Financial Implications, Psychiatria Danubina. (2020) 32, no. Suppl 4, 553–561. 33212463

[3] Liang S. E. , Cohen J. M. , and Ho R. S. , Screening for Depression and Suicidality in Psoriasis Patients: A Survey of US Dermatologists, Journal of the American Academy of Dermatology. (2019) 80, no. 5, 1460–1462, 10.1016/j.jaad.2019.01.025, 2-s2.0-85064275962. 30682396

[4] Wu J. J. , Kavanaugh A. , Lebwohl M. G. , Gniadecki R. , and Merola J. F. , Psoriasis and Metabolic Syndrome: Implications for the Management and Treatment of Psoriasis, Journal of the European Academy of Dermatology and Venereology. (2022) 36, no. 6, 797–806, 10.1111/jdv.18044. 35238067 PMC9313585

[5] Nowowiejska J. , Baran A. , and Flisiak I. , Aberrations in Lipid Expression and Metabolism in Psoriasis, International Journal of Molecular Sciences. (2021) 22, no. 12, 10.3390/ijms22126561, 6561. 34207318 PMC8234564

[6] Boehncke W.-H. and Schön M. P. , Psoriasis, The Lancet. (2015) 386, no. 9997, 983–994, 10.1016/S0140-6736(14)61909-7, 2-s2.0-84941421180. 26025581

[7] Wang J. , Yu Y. , and Liu L. , et al.Global Prevalence of Obesity in Patients With Psoriasis: An Analysis in the Past Two Decades, Autoimmunity Reviews. (2024) 23, no. 6, 10.1016/j.autrev.2024.103577, 103577. 39009055

[8] Jensen P. and Skov L. , Psoriasis and Obesity, Dermatology. (2017) 232, no. 6, 633–639, 10.1159/000455840, 2-s2.0-85013668802. 28226326

[9] Zhou D. , Pan Q. , and Shen F. , et al.Total Fecal Microbiota Transplantation Alleviates High-Fat Diet-Induced Steatohepatitis in Mice via Beneficial Regulation of Gut Microbiota, Scientific Reports. (2017) 7, no. 1, 10.1038/s41598-017-01751-y, 2-s2.0-85019222164, 1529. 28484247 PMC5431549

[10] Martin S. , Tyrrell J. , and Thomas E. L. , et al.Disease Consequences of Higher Adiposity Uncoupled From Its Adverse Metabolic Effects Using Mendelian Randomisation, eLife. (2022) 11, 10.7554/eLife.72452, 11. PMC8789289 35074047

[11] Thomas D. M. , Bredlau C. , and Bosy-Westphal A. , et al.Relationships Between Body Roundness With Body Fat and Visceral Adipose Tissue Emerging From a New Geometrical Model, Obesity. (2013) 21, no. 11, 2264–2271, 10.1002/oby.20408, 2-s2.0-84887206929. 23519954 PMC3692604

[12] Mahajan A. , Mahajan S. , and Tilekar S. , A Pilot Randomized Controlled Trial of Interval Training and Sleep Hygiene for Improving Sleep in Older Adults, Journal of Aging and Physical Activity. (2021) 29, no. 6, 993–1002, 10.1123/japa.2020-0207. 33837158

[13] Tian B.-W. , Yang Y.-F. , and Yang C.-C. , et al.Systemic Immune-Inflammation Index Predicts Prognosis of Cancer Immunotherapy: Systemic Review and Meta-Analysis, Immunotherapy. (2022) 14, no. 18, 1481–1496, 10.2217/imt-2022-0133. 36537255

[14] Xie R. , Xiao M. , and Li L. , et al.Association Between SII and Hepatic Steatosis and Liver Fibrosis: A Population-Based Study, Frontiers in Immunology. (2022) 13, 10.3389/fimmu.2022.925690, 925690. 36189280 PMC9520084

[15] Wang J. , Zhou D. , Dai Z. , and Li X. , Association Between Systemic Immune-Inflammation Index and Diabetic Depression, Clinical Interventions in Aging. (2021) 16, 97–105, 10.2147/CIA.S285000. 33469277 PMC7810592

[16] Tang Y. , Peng B. , Liu J. , Liu Z. , Xia Y. , and Geng B. , Systemic Immune-Inflammation Index and Bone Mineral Density in Postmenopausal Women: A Cross-Sectional Study of the National Health and Nutrition Examination Survey (NHANES) 2007–2018, Frontiers in Immunology. (2022) 13, 10.3389/fimmu.2022.975400, 975400. 36159805 PMC9493473

[17] Qin Z. , Li H. , and Wang L. , et al.Systemic Immune-Inflammation Index is Associated With Increased Urinary Albumin Excretion: A Population-Based Study, Frontiers in Immunology. (2022) 13, 10.3389/fimmu.2022.863640, 863640. 35386695 PMC8977553

[18] Griffiths C. E. M. , Armstrong A. W. , Gudjonsson J. E. , and Barker J. N. W. N. , Psoriasis, The Lancet. (2021) 397, no. 10281, 1301–1315, 10.1016/S0140-6736(20)32549-6. 33812489

[19] Zhao X. , Li J. , and Li X. , Association Between Systemic Immune-Inflammation Index and Psoriasis: A Population-Based Study, Frontiers in Immunology. (2024) 15, 10.3389/fimmu.2024.1305701, 1305701. 38504983 PMC10948528

[20] Yorulmaz A. , Hayran Y. , Akpinar U. , and Yalcin B. , Systemic Immune-Inflammation Index (SII) Predicts Increased Severity in Psoriasis and Psoriatic Arthritis, Current Health Sciences Journal. (2020) 46, no. 4, 352–357, 10.12865/chsj.46.04.05. 33717509 PMC7948012

[21] Ding Q. , Li X. , and Lin L. , et al.Association Between Systemic Immunity-Inflammation Index and Psoriasis Among Outpatient US Adults, Frontiers in Immunology. (2024) 15, 10.3389/fimmu.2024.1368727, 1368727. 38895126 PMC11183782

[22] Hu B. , Yang X.-R. , and Xu Y. , et al.Systemic Immune-Inflammation Index Predicts Prognosis of Patients After Curative Resection for Hepatocellular Carcinoma, Clinical Cancer Research. (2014) 20, no. 23, 6212–6222, 10.1158/1078-0432.CCR-14-0442, 2-s2.0-84918495553. 25271081

[23] Johnson C. L. , Paulose-Ram R. , and Ogden C. L. , et al.National Health and Nutrition Examination Survey: Analytic Guidelines, 1999–2010, Vital and Health Statistics. (2013) 2, 1–24. 25090154

[24] Bao W. , Liu B. , Simonsen D. W. , and Lehmler H.-J. , Association Between Exposure to Pyrethroid Insecticides and Risk of All-Cause and Cause-Specific Mortality in the General US Adult Population, JAMA Internal Medicine. (2020) 180, no. 3, 367–374, 10.1001/jamainternmed.2019.6019. 31886824 PMC6990752

[25] Menke A. , Casagrande S. , Geiss L. , and Cowie C. C. , Prevalence of and Trends in Diabetes Among Adults in the United States, 1988–2012, JAMA. (2015) 314, no. 10, 1021–1029, 10.1001/jama.2015.10029, 2-s2.0-84941702063. 26348752

[26] Liu C. , Liang D. , Xiao K. , and Xie L. , Association Between the Triglyceride-Glucose Index and All-Cause and CVD Mortality in the Young Population With Diabetes, Cardiovascular Diabetology. (2024) 23, no. 1, 10.1186/s12933-024-02269-0, 171. 38755682 PMC11097545

[27] Li C. and Shang S. , Relationship Between Sleep and Hypertension: Findings From the NHANES (2007–2014), International Journal of Environmental Research and Public Health. (2021) 18, no. 15, 10.3390/ijerph18157867, 7867. 34360157 PMC8345503

[28] Miao H. , Liu Y. , Tsai T. C. , Schwartz J. , and Ji J. S. , Association Between Blood Lead Level and Uncontrolled Hypertension in the US Population (NHANES 1999–2016), Journal of the American Heart Association. (2020) 9, no. 13, 10.1161/JAHA.119.015533. PMC7670543 32573312

[29] Chobanian A. V. , Bakris G. L. , and Black H. R. , et al.The Seventh Report of the Joint National Committee on Prevention, Detection, Evaluation, and Treatment of High Blood Pressure: The JNC 7 Report, JAMA. (2003) 289, no. 19, 2560–2572, 10.1001/jama.289.19.2560, 2-s2.0-0038460302. 12748199

[30] Wan Z. , Guo J. , Pan A. , Chen C. , Liu L. , and Liu G. , Association of Serum 25-Hydroxyvitamin D Concentrations With All-Cause and Cause-Specific Mortality Among Individuals With Diabetes, Diabetes Care. (2021) 44, no. 2, 350–357, 10.2337/dc20-1485. 33168652

[31] Tashiro T. and Sawada Y. , Psoriasis and Systemic Inflammatory Disorders, International Journal of Molecular Sciences. (2022) 23, no. 8, 10.3390/ijms23084457, 4457. 35457278 PMC9028262

[32] Barros G. , Duran P. , Vera I. , and Bermúdez V. , Exploring the Links Between Obesity and Psoriasis: A Comprehensive Review, International Journal of Molecular Sciences. (2022) 23, no. 14, 10.3390/ijms23147499, 7499. 35886846 PMC9321445

[33] Grundy S. M. , Cleeman J. I. , and Daniels S. R. , et al.Diagnosis and Management of the Metabolic Syndrome: An American Heart Association/National Heart, Lung, and Blood Institute Scientific Statement, Current Opinion in Cardiology. (2006) 21, no. 1, 1–6, 10.1097/01.hco.0000200416.65370.a0. 16355022

[34] Després J.-P. , Lemieux I. , and Prud’homme D. , Treatment of Obesity: Need to Focus on High Risk Abdominally Obese Patients, BMJ. (2001) 322, no. 7288, 716–720, 10.1136/bmj.322.7288.716. 11264213 PMC1119905

[35] Kumar P. , Monin L. , and Castillo P. , et al.Intestinal Interleukin-17 Receptor Signaling Mediates Reciprocal Control of the Gut Microbiota and Autoimmune Inflammation, Immunity. (2016) 44, no. 3, 659–671, 10.1016/j.immuni.2016.02.007, 2-s2.0-84960336674. 26982366 PMC4794750

[36] Snodgrass R. G. , Huang S. , Choi I.-W. , Rutledge J. C. , and Hwang D. H. , Inflammasome-Mediated Secretion of IL-1β in Human Monocytes Through TLR2 Activation; Modulation by Dietary Fatty Acids, The Journal of Immunology. (2013) 191, no. 8, 4337–4347, 10.4049/jimmunol.1300298, 2-s2.0-84885467630. 24043885 PMC3825708

[37] Huang S. , Rutkowsky J. M. , and Snodgrass R. G. , et al.Saturated Fatty Acids Activate TLR-Mediated Proinflammatory Signaling Pathways, Journal of Lipid Research. (2012) 53, no. 9, 2002–2013, 10.1194/jlr.D029546, 2-s2.0-84864829032. 22766885 PMC3413240

[38] Schwartz E. A. , Zhang W.-Y. , and Karnik S. K. , et al.Nutrient Modification of the Innate Immune Response, Arteriosclerosis, Thrombosis, and Vascular Biology. (2010) 30, no. 4, 802–808, 10.1161/ATVBAHA.109.201681, 2-s2.0-77950888339. 20110572

[39] Weber K. J. , Sauer M. , and He L. , et al.PPARγ Deficiency Suppresses the Release of IL-1β and IL-1α in Macrophages via a Type 1 IFN–Dependent Mechanism, The Journal of Immunology. (2018) 201, no. 7, 2054–2069, 10.4049/jimmunol.1800224, 2-s2.0-85053462921. 30143592 PMC6147147

[40] Kumar S. , Han J. , Li T. , and Qureshi A. A. , Obesity, Waist Circumference, Weight Change and the Risk of Psoriasis in US Women, Journal of the European Academy of Dermatology and Venereology. (2013) 27, no. 10, 1293–1298, 10.1111/jdv.12001, 2-s2.0-84884910431. 23057623 PMC4179884

[41] Paroutoglou K. , Papadavid E. , Christodoulatos G. S. , and Dalamaga M. , Deciphering the Association Between Psoriasis and Obesity: Current Evidence and Treatment Considerations, Current Obesity Reports. (2020) 9, no. 3, 165–178, 10.1007/s13679-020-00380-3. 32418186

[42] Carrascosa J. M. , Vilavella M. , and Garcia-Doval I. , et al.Body Mass Index in Patients With Moderate-to-Severe Psoriasis in Spain and Its Impact as an Independent Risk Factor for Therapy Withdrawal: Results of the Biobadaderm Registry, Journal of the European Academy of Dermatology and Venereology. (2014) 28, no. 7, 907–914, 10.1111/jdv.12208, 2-s2.0-84905028303. 23848131

[43] Habjanič N. , Lužar-Stiffler V. , Kerec-Kos M. , and Grabnar Peklar D. , Efficacy of Calcipotriol-Betamethasone Ointment in Patients With Mild to Moderate Plaque Psoriasis: Subgroup Analyses, Dermatology. (2019) 235, no. 6, 501–508, 10.1159/000502516, 2-s2.0-85072176188. 31509844

[44] Alruwaili H. , Dehestani B. , and le Roux C. W. , Clinical Impact of Liraglutide as a Treatment of Obesity, Clinical Pharmacology: Advances and Applications. (2021) 13, 53–60, 10.2147/CPAA.S276085. 33732030 PMC7958997

[45] Zhong P. , Zeng H. , Huang M. , Fu W. , and Chen Z. , Efficacy and Safety of Once-Weekly Semaglutide in Adults With Overweight or Obesity: A Meta-Analysis, Endocrine. (2022) 75, no. 3, 718–724, 10.1007/s12020-021-02945-1. 34981419

[46] Zobel E. H. , Ripa R. S. , and von Scholten B. J. , et al.Effect of Liraglutide on Expression of Inflammatory Genes in Type 2 Diabetes, Scientific Reports. (2021) 11, no. 1, 10.1038/s41598-021-97967-0, 18522. 34535716 PMC8448739

[47] Dinour L. , Leung M. M. , Tripicchio G. , Khan S. , and Yeh M.-C. , The Association Between Marital Transitions, Body Mass Index, and Weight: A Review of the Literature, Journal of Obesity. (2012) 2012, 10.1155/2012/294974, 2-s2.0-84867283452, 294974. 23050125 PMC3461301

[48] Lebwohl M. , Obesity, Smoking, and Psoriasis, JAMA. (2006) 295, no. 2, 208–210, 10.1001/jama.295.2.208, 2-s2.0-30444432567. 16715548

[49] Attwa E. and Swelam E. , Relationship Between Smoking-Induced Oxidative Stress and the Clinical Severity of Psoriasis, Journal of the European Academy of Dermatology and Venereology. (2011) 25, no. 7, 782–787, 10.1111/j.1468-3083.2010.03860.x, 2-s2.0-79955629224. 21039915

[50] Grundy S. M. , Stone N. J. , and Bailey A. L. , et al.2018 AHA/ACC/AACVPR/AAPA/ABC/ACPM/ADA/AGS/APhA/ASPC/NLA/PCNA Guideline on the Management of Blood Cholesterol: A Report of the American College of Cardiology/American Heart Association Task Force on Clinical Practice Guidelines, Circulation. (2019) 139, no. 25, e1082–e143, 10.1161/cir.0000000000000625, 2-s2.0-85068374636. 30586774 PMC7403606

[51] Greb J. E. , Goldminz A. M. , and Elder J. T. , et al.Psoriasis, Nature Reviews Disease Primers. (2016) 2, no. 1, 10.1038/nrdp.2016.82, 2-s2.0-84997207210, 16082. 27883001

[52] Bai G. , Peng Y. , and Liu Q. , et al.Association Between Body Roundness Index and Psoriasis Among US Adults: A Nationwide Population-Based Study, Lipids in Health and Disease. (2024) 23, no. 1, 10.1186/s12944-024-02365-w, 373. 39538202 PMC11559072

[53] Caballero B. , Humans Against Obesity: Who Will Win?, Advances in Nutrition. (2019) 10, no. suppl_1, S4–9, 10.1093/advances/nmy055, 2-s2.0-85061149716. 30721956 PMC6363526

[54] Aune D. , Snekvik I. , Schlesinger S. , Norat T. , Riboli E. , and Vatten L. J. , Body Mass Index, Abdominal Fatness, Weight Gain and the Risk of Psoriasis: A Systematic Review and Dose-Response Meta-Analysis of Prospective Studies, European Journal of Epidemiology. (2018) 33, no. 12, 1163–1178, 10.1007/s10654-018-0366-z, 2-s2.0-85045730623. 29680995 PMC6290660

[55] Budu-Aggrey A. , Brumpton B. , and Tyrrell J. , et al.Evidence of a Causal Relationship Between Body Mass Index and Psoriasis: A Mendelian Randomization Study, PloS Medicine. (2019) 16, no. 1, 10.1371/journal.pmed.1002739, 2-s2.0-85060952703. PMC6354959 30703100

[56] Schweitzer K. , Could the Body Roundness Index One Day Replace the BMI?, JAMA. (2024) 332, no. 16, 10.1001/jama.2024.20115, 1317. 39365577

[57] Ghoreschi K. , Balato A. , Enerbäck C. , and Sabat R. , Therapeutics Targeting the IL-23 and IL-17 Pathway in Psoriasis, The Lancet. (2021) 397, no. 10275, 754–766, 10.1016/S0140-6736(21)00184-7. 33515492

